# A Comparison of the Functional Traits of Common Reed (*Phragmites australis*) in Northern China: Aquatic vs. Terrestrial Ecotypes

**DOI:** 10.1371/journal.pone.0089063

**Published:** 2014-02-19

**Authors:** Liping Li, Wenxuan Han, Niels Thevs, Xiuhong Jia, Chengjun Ji, Dongmei Jin, Ping He, Armin O. Schmitt, Giuseppe Tommaso Cirella, Stefan Zerbe

**Affiliations:** 1 Key Laboratory of Bioactive Substances and Resources Utilization of Chinese Herbal Medicine (Peking Union Medical College), Ministry of Education, Institute of Medicinal Plant Development, Chinese Academy of Medical Sciences, Peking Union Medical College, Beijing, China; 2 Faculty of Science and Technology, Free University of Bozen-Bolzano, Bolzano, Italy; 3 Key Laboratory of Plant-Soil Interactions, Ministry of Education, College of Resources and Environmental Sciences, China Agricultural University, Beijing, China; 4 Institute of Botany and Landscape Ecology, University of Greifswald, Greifswald, Germany; 5 College of Horticulture and Forestry, Huazhong Agricultural University, Wuhan, China; 6 Department of Ecology, College of Urban and Environmental Sciences, Key Laboratory for Earth Surface Processes of the Ministry of Education, Peking University, Beijing, China; 7 State Environmental Protection Key Laboratory of Regional Eco-Process and Function Assessment, Chinese Research Academy of Environmental Sciences, Beijing, China; University of Vigo, Spain

## Abstract

Common reed (*Phragmites australis* (Cav.) Trin. ex Steud.) is distributed widely throughout the world with various ecotypes. This research compares the functional traits and biomass allocation patterns of two contrasting reed ecotypes. Twelve pairs of aquatic and terrestrial reed samples were collected in northern China. Significant differences in functional traits between the two reed ecotypes were observed, while biomass allocation patterns of reed organs did not differ significantly except for at the root. The dry matter content (DMC) in the whole of the reed plant, leaf, root, and rhizome was higher; while the specific leaf area (SLA) and specific root length (SRL) were lower in terrestrial versus aquatic reed. The biomass allocation in organs of the two forms of reed was isometric, only root in the terrestrial habitat increased faster with an increase in the whole plant biomass. It can be affirmed that aquatic and terrestrial reed that adapt to different environments generally has distinct leaf and root functional traits but isometric biomass allocation patterns. This suggests different resource acquisition strategies: (1) aquatic reed grows faster with high SLA and SRL and is more responsive to the environment, while (2) terrestrial reed with high DMC grows slower and is less responsive to the adverse environment (e.g. dry soil conditions).

## Introduction

It is of significant interest, within the field of plant ecology, the study of the relationships between plant functional traits [1]. As two of the most active metabolic organs, root and leaf are very important for plant functioning [2]. Their traits are associated closely with carbon and nutrient uptake and plant growth rates [3]. Compared to slow-growing species, plants with high growth rates usually have large specific root length (SRL), small root diameter, large specific leaf area (SLA), low dry matter content (DMC), low tissue longevity, low nutrient use efficiency and, generally, are better adapted to nutrient rich environments [4], [5]. Leaf and root traits are functionally associated [6], as well as associated with other organs (e.g. stem and rhizome) [7]–[9]. Trait analyses that integrate leaf, stem and root across gradients of soil fertility and water availability are baseline for future research [8], [10].

Biomass allocation patterns in different plant organs (e.g. flower, leaf, stem, root, and rhizome) can also reflect trade-offs between functional traits. Changes in plant biomass allocation patterns were considered phenotypic plasticity or allometric strategies [11]. Some studies found stable, i.e. isometric biomass allocation patterns [12]–[16], while others concluded that the allocation patterns were not stable and would change with plant growth and size (i.e. optimal partitioning) [17], [18]. Meta-analysis showed that plants would alter the organ morphology rather than adjust the biomass allocations [9]. Large scale research conducted in the Tibetan grasslands of China found the isometric biomass allocation patterns [14], [15]; moreover, the isometric patterns over time have been found globally throughout related forest ecosystems [16].

Plant functional traits were mainly studied in terrestrial plants (e.g. [19]–[21]); however, terrestrial and aquatic plants grow in completely differing environments and may face totally different stresses. For example, terrestrial plants are stressed more frequently by water shortage, while aquatic plants may often suffer from long-term oxygen deprivation [22]. The contrasting environmental stresses on aquatic and terrestrial plants supposedly can cause the differing in functional traits. Thus, the comparison of functional traits between plants in terrestrial and aquatic habitats may lead to a better understanding of the trade-offs between the two. Former studies found that there were big phenotypic differences between *Polygonum lapathifolium* when this plant was planted in dry and flooded soils, respectively [23].

Common reed (*Phragmites australis* (Cav.) Trin. ex Steud.) has wide distribution ranges throughout the world with regard to ground water level, nutrient supply, soil-water salinity, and land-use [24]–[26]. *P. australis* could also be a bio-indicator since its morphology changes with the growing environment [27]. It is an ideal plant for comparing functional traits in different environments. With twelve pairs of aquatic and terrestrial samples taken from the Wuliangsuhai Lake area and the wetlands in the Zhangye city in China, functional traits and biomass allocation patterns between the two ecotypes of *P. australis*, aquatic and terrestrial, were compared. The aim is to test whether there are differences in the functional traits (DMC of different organs, SLA, SRL, mean root diameter, and mean root area of unit mass) between aquatic and terrestrial reed. A further investigation is to find out if the organ biomass allocations of aquatic and terrestrial reed follow the optimal partitioning strategy or if it is isometric.

## Materials and Methods

### Study Sites

The study sites were located in northern China near the Wuliangsuhai Lake of Inner Mongolia and the wetlands in the Zhangye city, Gansu Province. Wuliangsuhai Lake is located around N41° and E109° and Zhangye N39° and E100°. The mean annual temperature in both areas is 7°C, while the mean annual precipitation is 216 mm in the Wuliangsuhai Lake area and 129 mm in the Zhangye city [28], [29]. Wuliangsuhai Lake is an important wetland area in the Hetao Irrigation District watershed which flows into the Yellow River. Approximately 4.5∼7.0 billion m^3^ of annual water, within the forty year block of the 1960s to 1990s [30], from Yellow River goes into the Hetao Irrigation District watershed area; this traditionally has satisfied the water demand of crops and has kept the water level of Wuliansuhai Lake relatively stable. Wetlands in Zhangye are closely connected with the Hei River.

At both sites *P. australis* is widely spread as the dominant emergent species. Near the water habitat, *P. australis* grows with relatively lower stem density and stem height along extreme arid land. The locations of the two sites in China were mapped and the environmental conditions were recorded in reference [31].

### Field Sampling and On-site Analysis

In August 2011, the reed plants were sampled in pairs, at the same spot, one in water habitat and the other on land. At this time of the year reed plants are at their peak biomass levels. Sampling sites were chosen in terms of representative reed communities with little competition from other species. We defined *P. australis* sampling within the two habitats as aquatic reed and terrestrial reed, respectively. Covering a wide environmental range, five pairs were sampled in the Wuliangsuhai Lake area and seven pairs in the wetlands around the Zhangye city (Table 1). The sampling work was supported by the local government that was Hetao Water Affairs Group, Inner Mongolia and Zhangye Environment Protection Bureau, Gansu. Mature and well-grown individuals were sampled. For water habitat, we chose *P. australis* individuals that had been established in a water body of more than 10 cm in depth, while for terrestrial habitat reed, *P. australis* individuals were within 10 m in distance from the water body source. The water habitat was saturated while the terrestrial one was not. Moreover, for terrestrial habitat the soil water content was very low due to a large number of individuals living along a bankside water source. In both sampled regions, potential evapotranspiration (PET) was very high [28], [29]. Belowground parts were sampled 30 cm below the sediment or soil surface, with around a 10 cm radius from the main stem circle-center. Each individual was dissected into leaf, stem, flower, fine root (termed root hereafter), and rhizome. The fresh biomass of the five organs was weighed in the field immediately after sampling. Subsequently, a part of the five organs was brought to the laboratory for further analysis. In addition, sediment and water in the aquatic habitat and soil in the terrestrial habitat were collected and analyzed near the plant samples, in order to supply a background setting of the environmental conditions for the sampling sites. At least three samples were collected for each and mixed together for the further analysis. Both water electric conductivity (EC, ms cm^−1^) and pH were measured on site with a Multi340i handheld meter (WTW, Weilheim, Germany).

**Table 1:** Characteristics of sediment, soil, and water in terrestrial and aquatic habitats.

	Aquatic habitat	Terrestrial habitat
Sediment/soil EC (ms cm^−1^)	1.09^a^±0.63	2.22^b^±1.63
Sediment/soil pH	7.45^a^±0.18	7.49^a^±0.32
Sediment/soil TN (mg g^−1^)	1.13^a^±0.83	0.56^a^±0.36
Sediment/soil TP (mg g^−1^)	0.69^a^±0.25	0.52^a^±0.16
Water EC (ms cm^−1^)	2.83±4.54	–
Water pH	8.28±0.75	–
Water TN (mg g^−1^)	4.39±5.03	–
Water TP (mg g^−1^)	0.026±0.068	–

Shown are the geometric means and the standard deviations (SD). The different superscripts indicate significant differences in the means (paired t-test, *P*<0.05).

### Lab Analysis

A total ranging from 10–30 leaves and 0.5 g wet root (1–5 replicates) were scanned, with a Canon scanner (4400F), per sample. Then SLA, SRL, mean root diameter (R_diam_) and mean root area of unit mass (R_area_) were determined with WinFOLIA and WinRHIZO (Régent, Quebec, Canada). All samples were oven-dried at 60°C for 72 h to get the value of DMC. Sediment EC and pH were determined with a DDS-11A digital display conductivity meter (CSDIHO, Shanghai, China) and PH-3C (INESA, Shanghai, China). Total nitrogen (TN, mg g^−1^) in the sediment and water was measured according to the Alkaline potassium persulfate digestion-UV spectro-photo-metric method [32]. Total phosphorous (TP, mg g^−1^) in the sediment and water was measured following the molybdate stannous chloride method [32].

### Data Analysis

We compared the functional traits of *P. australis* from both study sites and found no significant differences. Hence, we pooled together the data from the two study sites for further analyses. Since the sampling sites of the terrestrial and the aquatic habitat were in close vicinity (less than 10 m) we expected no other variations due to environmental conditions and therefore used the paired t-test to test for the differences in the traits of *P. australis* in the two habitats, as well as for characteristic differences between the two habitats. One-sided paired t-tests were used for DMC, SLA, SRL, R_area_ and R_diam_. DMC and R_diam_ were expected to be higher in terrestrial reed and SLA, SRL, and R_area_ to be higher in aquatic reed. Two-sided paired t-tests were used to test for any differences in biomass allocation in *P. australis* organs (i.e. flower, leaf, stem, root, and rhizome), and Root:Shoot ratio (RS). In this case, the proportion of root to the total biomass was also defined as Root Ratio (RR). The results of ANOVA for these characteristics of the two reed ecotypes were presented in Table S1.

Standardized major axis (SMA) analysis was used to detect relationships between biomass allocations in different organs. Slopes of SMA were compared between the two reed ecotypes [21]. We considered more the trait differences of *P. australis* due to the two types of habitats (mostly moisture differences) and ignored the differences of other environmental variables, for example, soil/sediment nutrient contents.

All analyses were carried out in R 2.12.2 [33]. The SMA analyses were performed with the ‘smatr’ package [34].

## Results

### Dry Matter Content (DMC) of Aquatic and Terrestrial Reed

**Figure 1:**
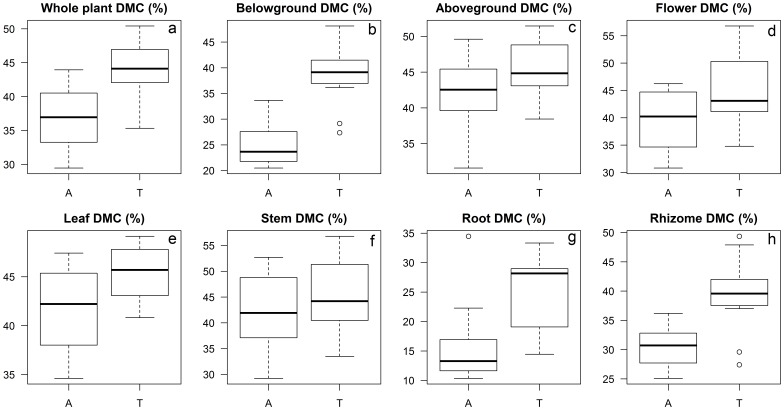
The dry matter content (DMC) of aquatic (A) and terrestrial (T) reed for the (a) whole plant, (b) belowground part, (c) aboveground part and (d–h) separate organs (the solid lines in the boxes indicate median values, the upper and lower ranges of the boxes show the third and first quartiles whereas upper and lower lines out of the boxes indicate the maximum and minimum values, extreme values are shown as dots, analogously for Figure 2).

The mean DMC (mean ± standard deviation, SD) ranged from 15.7%±6.8% in root to 42.4%±7.5% in stem of aquatic reed, and from 25.1%±6.5% in root to 45.8%±2.8% in leaf of terrestrial reed (Figure 1). The DMC was higher in terrestrial than in aquatic reed. This held true for both the whole plant and its parts (i.e. belowground part, leaf, root, and rhizome, *P*<0.05, Figure 1a,b,e,g,h). The DMC of the aboveground part was slightly higher for terrestrial than for aquatic reed (*P*<0.1, Figure 1c). There were no significant differences in DMC of flower and stem between the two forms (*P*>0.1, Figure 1d,f).

### Leaf and Root Traits of Aquatic and Terrestrial Reed

**Figure 2:**
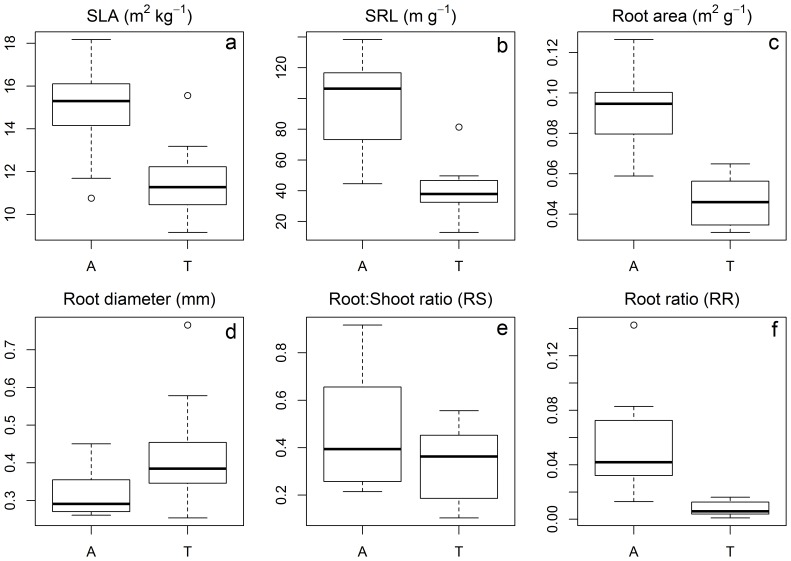
The functional traits and biomass allocations of aquatic (A) and terrestrial (T) reed (a, specific leaf area (SLA); b, specific root length (SRL); c, mean root area of unit mass (R_area_); d, mean root diameter (R_diam_); e, Root:Shoot ratio (RS); f, root ratio (RR)).

The mean SLA (mean ± SD) were 14.9±2.0 and 11.7±1.8 m^2^ kg^−1^ and the mean SRL for aquatic and terrestrial reed were 94.9±29.2 and 40.4±18.1 m g^−1^, respectively. The SLA and SRL were both higher for aquatic than for terrestrial reed (*P*<0.05, Figure 2a,b). The mean root area of unit mass (R_area_) was greater but the mean root diameter (R_diam_) was smaller for aquatic than for terrestrial reed (R_area_ = 0.09±0.02 and 0.05±0.01 m^2^ g^−1^; and R_diam_ = 0.32±0.07 and 0.42±0.14 mm for aquatic and terrestrial reed, respectively, *P*<0.05, Figure 2c,d).

### Biomass Allocations of Aquatic and Terrestrial Reed

The biomass proportions (dry weight) of the aboveground part, flower, leaf, stem, and rhizome did not differ significantly between the aquatic and terrestrial ecotypes (70% and 76% for aboveground biomass proportions, 3.9% and 8.9% for flower, 29.5% and 36.2% for leaf, 39.5% and 32.3% for stem, 21.4% and 21.9% for rhizome, for aquatic and terrestrial reed, respectively, all *P*>0.05, Table 2). The mean RS did not differ significantly between aquatic and terrestrial reed (0.45 and 0.32, *P*>0.05, Table 2 and Figure 2e); however, the biomass proportion of root was significantly higher for aquatic than for terrestrial reed (5.5% and 0.7%, *P*<0.05, Table 2 and Figure 2f).

**Table 2:** Biomass allocations of aquatic and terrestrial reed ecotypes.

	(%)	Flower	Leaf	Stem	Root*	Rhizome
Aquaticecotype	Mean	3.9^a^	29.5^a^	39.5^a^	5.5^a^	21.4^a^
	SD	2.9	9.5	9.6	3.5	7.0
Terrestrialecotype	Mean	8.9^a^	36.2^a^	32.3^a^	0.7^b^	21.9^a^
	SD	5.9	8.1	8.5	0.6	8.0
		RS	Leaf:Stem	Root:Rhizome	Root:Leaf	Stem:Rhizome
Aquaticecotype	Mean	0.45^a^	0.83^a^	0.26^a^	0.22^a^	2.09^a^
	SD	0.24	0.47	0.13	0.18	0.95
Terrestrialecotype	Mean	0.33^a^	1.28^a^	0.04^b^	0.02^b^	1.84^a^
	SD	0.15	0.79	0.02	0.02	1.22

Leaf:Stem indicates the biomass ratio of leaf and stem, analogously for the Root:Rhizome, Root:Leaf, and Stem:Rhizome. The different superscripts indicate significant differences in the means (paired t-test, *P*<0.05). SD, standard deviation.

*The proportion of root biomass is also defined as root ratio (RR).

**Figure 3:**
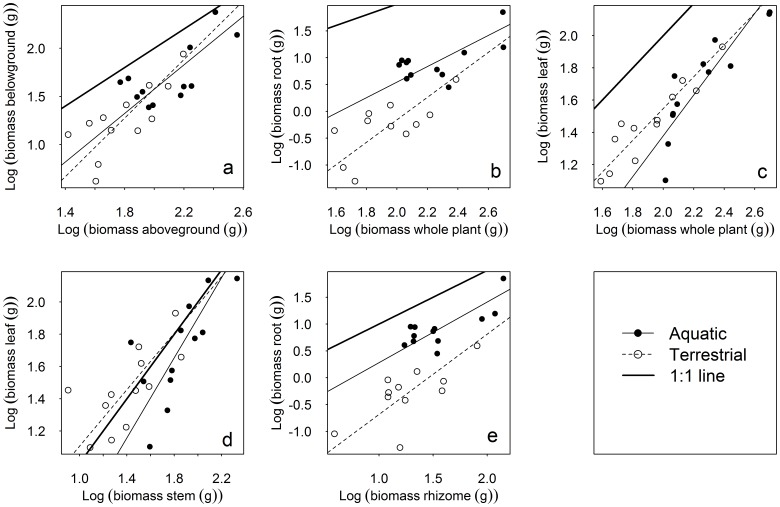
The biomass allocation patterns between organs and the belowground/whole plant of *P. australis* (a, aboveground and belowground biomass; b, root and whole plant biomass; c, leaf and whole plant biomass; d, leaf and stem biomass; e, root and rhizome biomass; with data log_10_-transformed).

There was positive correlation between biomass of aboveground and belowground for both terrestrial and aquatic reed (*P*<0.05). The slopes for the two regressions were not significantly different and neither were significantly different from 1.0 (SMA regression, *P*>0.05, Table 3 and Figure 3a). The positive correlation also existed between leaf/root biomass and the whole plant biomass in which the SMA slopes for the two ecotypes were not significantly different (Table 3 and Figure 3b,c). The slope of the root against whole plant biomass of the aquatic form did not differ significantly from 1.0 while the corresponding slope of the terrestrial form was significantly greater than 1.0 (Table 3 and Figure 3b). The slope of the leaf against whole plant biomass of both forms did not differ significantly from 1.0 (Table 3 and Figure 3c).

**Table 3:** The relationships of biomass allocation in different *P. australis* organs in the aquatic and terrestrial ecotypes (data were log_10_-transformed).

	Ecotype	Slope	Lower	Upper	*r^2^*	*P*
Biomass below-and aboveground	Aquatic	1.26	0.78	2.04	0.49	0.01
	Terrestrial	1.52	0.97	2.37	0.58	<0.01
Biomass rootand whole plant	Aquatic	1.45	0.86	2.46	0.39	0.03
	Terrestrial	2.10	**1.24**	**3.55**	0.46	0.02
Biomass leafand whole plant	Aquatic	1.26	0.91	1.77	0.77	<0.01
	Terrestrial	0.99	0.76	1.28	0.86	<0.01
Biomass leafand stem	Aquatic	1.24	0.78	1.99	0.52	<0.01
	Terrestrial	0.88	0.54	1.43	0.47	0.01
Biomass rootand rhizome	Aquatic	1.13	0.72	1.76	0.57	<0.01
	Terrestrial	1.50	0.89	2.53	0.47	0.02

Lower and upper indicate the 95% confidence interval (CI) of the SMA regression slope.

The ratio of root vs. rhizome biomass was greater for aquatic than for terrestrial reed (0.22 and 0.02, *P*<0.05, Table 2), illustrating that one unit of mass of rhizome supported more root in aquatic than in terrestrial reed. The ratio of leaf vs. stem biomass was slightly higher for terrestrial than for aquatic reed (*P*<0.1, Table 2) indicating that one unit of mass of stem appeared to support slightly greater leaf biomass in terrestrial than in aquatic reed. However, the slopes of the SMA regressions were not significantly different for both leaf vs. stem and root vs. rhizome from the two identified forms (Table 3 and Figure 3d,e). In addition, the slopes of the two regressions did not differ from 1.0, possibly suggesting the isometric scaling of leaf vs. stem biomass and root vs. rhizome biomass in the two reed ecotypes.

## Discussion

### Dry Matter Content

When dealing with plant traits, DMC is one of the most important indicators for resource use [35]. Leaf DMC is often negatively correlated with leaf growth [36]. An example of this relates to annual plants with higher growth rates having lower leaf DMC than perennial herb plants [37]. The organ DMC of terrestrial reed is significantly higher than aquatic reed in leaf, root, and rhizome, suggesting that aquatic reed with low DMC has a higher growth rate than terrestrial reed. In contrast, terrestrial reed, developed on land, grows slower and most likely resists better environmental stresses (e.g. frequent water shortage) due to the effect of high DMC.

### Leaf and Root Traits

Plants with high growth rates have high SLA, for example, annual plants have higher SLA than perennial herb plants [37]. Relatively low SLA under water shortage can be beneficial because this means that water use efficiency is enhanced [38]. It was found that plant SLA is lower in desert steppe (with lower water availability) than in meadow steppe and typical steppe [5]. Terrestrial reed has low SLA, i.e. it is adapted to adverse environments; for example it grows slower to cope with low water availability and soil salinization problems (terrestrial habitats have higher soil EC than that of sediment in aquatic habitat, Table 1). In contrast, aquatic reed that has high SLA which grows and expands faster. Low light availability caused by high stem density in aquatic habitat could also lead to high SLA of aquatic reed. Finally, aquatic reed that has high SRL, high R_area_ and low R_diam_, suggests a higher growth rate and nutrient use efficiency.

Species with high maximal relative growth rates occupy fertile habitat and are more sensitive to environmental nutrient changes [3]. It was found that competition decreased biomass of *Myriophyllum spicatum* in the fertile sediment more than in the infertile sediment [39]. But the potentially slow-growing species exhibited a higher phenotypic plasticity than the potentially fast-growing species when they were planted in soils with different nitrate contents [40], i.e. potentially slow-growing species changed the morphology more than fast-growing species to adapt to the change in soil nutrient availability. In this study, the aquatic reed is supposed to be more sensitive to environmental changes than terrestrial reed considering that the variations of both SLA and SRL are higher for aquatic than for terrestrial reed (Figure 2). This could be due to the sediment TN and TP (in which varying water pollution levels exist) of aquatic reed was highly variable, compared to terrestrial reed in which samples grew primarily on extremely arid and salinized soils where no other primary species grew.

### Biomass Allocation

Biomass allocation has profound implications for plant growth [17]. According to the hypothesis of optimal partitioning, decreased nutrient supply can increase biomass allocation to roots while decreased irradiance can increase biomass allocation to leaves [17]. The hypothesis suggests that plant biomass allocation (e.g. RS) is determined by factors that influence environmental nutrient, water and light availability and the allocation patterns possibly can change with varying environmental factors. Plants with low RS ratio are usually expected to grow in environments that are rich in nutrient and water availability [11] (i.e. RS is higher in nutrient- or water-stressed than in the more favorable environments for terrestrial plants). The hypothesis of isometric allocation suggests stable biomass allocation patterns that are insensitive to environmental conditions [13]. Isometric scaling was found at different scales and in various vegetation types, such as between aboveground and belowground biomass in China’s grasslands [14], [15], and over time in temporal forest ecosystems [16]. With a meta-analysis, it was found that woody and herbaceous taxa had different nutrient contents, while the statistical scaling did not differ both within and across organs [1]. In 2000, Müller et al. investigated the biomass allocations in 27 clonal species via different soil nutrient availabilities and found a similar scaling law in 21 species, albeit the scaling exponents were not equal to one [11]. Although there were some significant differences, (i.e. saturated and extreme arid of the two habitats) (Table 1), we did not find significant differences in RS between aquatic and terrestrial reed. The biomass allocation in different reed organs, except for root, did not differ significantly between the two forms of reed, indicating a generally similar biomass allocation pattern in the two reed ecotypes.

## Conclusion

With twelve pairs of samples, this study compared the functional traits and biomass allocation patterns of *P. australis* in aquatic and terrestrial habitats. We conclude that aquatic and terrestrial reed, established in contrasting environments, have generally similar biomass allocation patterns but distinct leaf and root functional traits which suggests different resource acquisition strategies, i.e. aquatic reed grows faster due to high SLA and SRL and is more responsive to the environment, while terrestrial reed grows slower and could resist more adverse environment due to the high DMC and may be less responsive to the environment at large.

The findings of this study could have implications for better understanding trade-offs of plant functional traits in changing climatic conditions. Climate change will become increasingly pronounced [41], especially for high latitude areas, like in northern China, where there has been a strong warming in recent years [42]. Water plants are more sensitive to the impacts of climatic change than terrestrial plants; recent research suggested a vast loss of species and community restructuring of wetland regions in the last four decades [43]. To better understand this impact, differences in the responses of functional traits of water and terrestrial plants to environmental change are important for predictive and scenario-based learning. *P. australis* dominated wetlands may have a considerable effect on and be affected by climatic change as it is species that is extensively found throughout the planet [44]. *P. australis* could be a model plant to exemplify wetland ecosystem changes in a dynamic global climate. Further studies, including genetics, are also needed to clearly understand different syndromes that correspond to the relating ecotypes.

## Supporting Information

Table S1 The ANOVA results of characteristics of environmental variables, dry matter content, functional traits, biomass allocations, and allometry of *P. australis* in aquatic and terrestrial habitat.
(DOCX)
