# Supplementary material for: A Comparison of the Functional Traits of Common Reed (Phragmites australis) in Northern China: Aquatic vs. Terrestrial Ecotypes
Source: PLoS One. 2014 Feb 19;9(2):e89063. doi: 10.1371/journal.pone.0089063 (PMC3929632; doi:10.1371/journal.pone.0089063)
Supplement: Table S1 — The ANOVA results of characteristics of environmental variables, dry matter content, functional traits, biomass allocations, and allometry of P. australis in aquatic and terrestrial habitat. (DOCX) [file pone.0089063.s001.docx]

Table S1. The ANOVA results of characteristics of environmental variables, functional traits, biomass allocations, and allometry of *Phragmites australis* in aquatic and terrestrial habitat*.*

|  | | *df* | *SS%* | *F* | *P* |
| --- | --- | --- | --- | --- | --- |
| **Environmental variables** | |  |  |  |  |
| pH | | 1 | 0.71 | 0.14 | 0.72 |
| Residuals | | 19 | 99.29 |  |  |
| EC | | 1 | 26.05 | 6.69 | 0.02 |
| Residuals | | 19 | 73.95 |  |  |
| TN | | 1 | 15.40 | 3.46 | 0.08 |
| Residuals | | 19 | 84.60 |  |  |
| TP | | 1 | 13.95 | 3.08 | 0.10 |
| Residuals | | 19 | 86.05 |  |  |
| **Dry matter content** | | |  |  |  |
| WDMC | 1 | | 37.05 | 12.95 | < 0.01 |
| Residuals | 22 | | 62.95 |  |  |
| WBDMC | 1 | | 66.37 | 43.42 | < 0.01 |
| Residuals | 22 | | 33.63 |  |  |
| WADMC | 1 | | 11.90 | 2.97 | 0.10 |
| Residuals | 22 | | 88.10 |  |  |
| FDMC | 1 | | 14.15 | 2.47 | 0.14 |
| Residuals | 15 | | 85.85 |  |  |
| LDMC | 1 | | 22.54 | 6.40 | 0.02 |
| Residuals | 22 | | 77.46 |  |  |
| SDMC | 1 | | 3.64 | 0.83 | 0.37 |
| Residuals | 22 | | 96.36 |  |  |
| RDMC | 1 | | 35.09 | 11.36 | < 0.01 |
| Residuals | 21 | | 64.91 |  |  |
| RHDMC | 1 | | 44.45 | 17.60 | < 0.01 |
| Residuals | 22 | | 55.55 |  |  |
| **Functional traits** | | |  |  |  |
| SLA | 1 | | 47.12 | 19.61 | < 0.01 |
| Residuals | 22 | | 52.88 |  |  |
| SRL | 1 | | 57.52 | 25.72 | < 0.01 |
| Residuals | 19 | | 42.48 |  |  |
| Rdiam | 1 | | 19.78 | 4.93 | 0.04 |
| Residuals | 20 | | 80.22 |  |  |
| Rarea | 1 | | 66.34 | 37.44 | < 0.01 |
| Residuals | 19 | | 33.66 |  |  |
| RS | 1 | | 8.70 | 2.10 | 0.16 |
| Residuals | 22 | | 91.30 |  |  |
| **Biomass allocations** | | |  |  |  |
| ProFlower | 1 | | 19.44 | 3.14 | 0.10 |
| Residuals | 13 | | 80.56 |  |  |
| ProLeaf | 1 | | 13.44 | 3.42 | 0.08 |
| Residuals | 22 | | 86.56 |  |  |
| ProStem | 1 | | 14.68 | 3.78 | 0.06 |
| Residuals | 22 | | 85.32 |  |  |
| ProRoot | 1 | | 47.42 | 18.95 | < 0.01 |
| Residuals | 21 | | 52.58 |  |  |
| ProRhizome | 1 | | 0.16 | 0.04 | 0.85 |
| Residuals | 22 | | 99.84 |  |  |
| **Allometry** |  | |  |  |  |
| LS | 1 | | 11.97 | 2.99 | 0.10 |
| Residuals | 22 | | 88.03 |  |  |
| RRH | 1 | | 57.84 | 28.82 | < 0.01 |
| Residuals | 21 | | 42.16 |  |  |
| RL | 1 | | 38.14 | 12.95 | < 0.01 |
| Residuals | 21 | | 61.86 |  |  |
| SRH | 1 | | 1.43 | 0.32 | 0.58 |
| Residuals | 22 | | 98.57 |  |  |

EC: Electric conductivity (ms cm^−1^) of soils or sediments; pH: pH of soils or sediments; TN: Total nitrogen (mg g^−1^) content of soils or sediments; TP: Total phosphorous (mg g^−1^) content of soils or sediments; WDMC: Dry matter content (%) of the whole plant; WBDMC: Dry matter content (%) of the belowground part; WADMC: Dry matter content (%) of the aboveground part; FDMC: Dry matter content (%) of flower; LDMC: Dry matter content (%) of leaf; SDMC: Dry matter content (%) of stem; RDMC: Dry matter content (%) of root; RHDMC: Dry matter content (%) of rhizome; SLA: Specific leaf area (m^2^ kg^-1^); SRL: Specific root length (m g^-1^); Rdiam: Mean root diameter (mm); Rarea: Mean root area (m^2^ g^-1^); RS: Biomass ratio of root: shoot; ProFlower: Flower biomass proportion (%); ProLeaf: Leaf biomass proportion (%); ProStem: Stem biomass proportion (%); ProRoot: Root biomass proportion (%); ProRhizome: Rhizome biomass proportion (%); LS: Biomass ratio of leaf: stem; RRH: Biomass ratio of root: rhizome; RL: Biomass ratio of root: leaf; SRH: Biomass ratio of stem: rhizome.
